# Therapeutic analysis of Herbert screw fixation for capitellar fractures via the anterior approach in adolescent patients

**DOI:** 10.1186/s13018-021-02536-w

**Published:** 2021-06-19

**Authors:** Lingpeng Ju, Linjun Jiang, Yuan Zhang, Jun Wu, Ming Li, Xing Liu, Xiangyang Qu

**Affiliations:** Department of Orthopedics, National Clinical Research Center for Child Health and Disorders, Ministry of Education Key Laboratory of Child Development and Disorders, Chongqing Key Laboratory of Pediatrics, Children’s Hospital of Chongqing Medical University, No. 136 of Zhong Shan Er Lu, Chongqing, 400014 China

**Keywords:** Capitellar fractures, Adolescents, Herbert screw, Anterior approach, Treatment

## Abstract

**Objective:**

The aim of this study is to analyze the efficacy of open reduction and Herbert screw fixation for coronal fractures of the capitellum via the anterior approach in adolescents.

**Methods:**

We retrospectively analyzed the clinical and imaging data of 15 adolescents with capitellar fractures who were admitted to our hospital from May 2014 to May 2019. The fracture was reduced through the cubital crease incision via the anterior approach and was internally fixated with Herbert screws. A follow-up was conducted after the operation to examine fracture healing and elbow function. The postoperative functional recovery of patients was evaluated with the Mayo Elbow Performance index (MEPI) and the Broberg-Morrey rating system.

**Results:**

Patients underwent surgery 3.7 days after injury on average. Intraoperative fracture reduction was satisfactory. No vascular injury or nerve injury occurred. Bony union occurred in an average of 6 weeks after the operation. All adolescents completed a 12- to 36-month follow-up. At the last follow-up, the Mayo Elbow Performance index was considered excellent in 12 patients and good in three patients. The Broberg-Morrey score was considered excellent in 12 patients, good in two patients, and fair in one patient.

**Conclusion:**

Open reduction with Herbert screw fixation via the anterior approach is a feasible surgical method for the treatment of coronal fractures of the capitellum in adolescents.

**Levels of evidence:**

Therapeutic, retrospective study-Level IV

## Introduction

Capitellar fracture in children is rare, accounts for approximately 0.5–1% of elbow fractures in children [1, 2]. Because the capitellum is located deep in the joint capsule, capitellar fracture in children typically does not cause apparent deformity in the elbow but manifests as swelling, pain, and dysfunction of the elbow joint. Anteroposterior or lateral radiography of the elbow joint cannot accurately diagnose all types of capitellar fractures. Therefore, the missed diagnosis and misdiagnosis rates of capitellar fractures in children are high [3]. Oblique radiography, computed tomography (CT), magnetic resonance imaging (MRI), and arthrography of the elbow joint are very important for the diagnosis of capitellar fracture in children [1, 3, 4].

Open reduction and internal fixation are the main approaches used to treat displaced capitellar fractures [1]. Capitellar fractures in children are intra-articular fractures. These fractures are prone to complications such as heterotopic ossification, avascular necrosis of bone, elbow stiffness, and posttraumatic arthritis [5]. Restoring the anatomical structure of the articular surface is very important to reduce the occurrence of posttraumatic arthritis [6]. The purpose of open reduction and internal fixation is to reconstruct the anatomical structure of the capitellum, restore the smoothness and flatness of the articular surface, and provide firm fixation to promote fracture healing and to restore elbow function.

To date, no unified opinion exists on the surgical approaches and fixation selection for the treatment of capitellar fractures [1, 5, 7, 8]. The lateral and posterior approaches are the most commonly used surgical methods for elbow joint surgery [9], but for the treatment of capitellar fractures, the lateral and posterior approaches provide poor exposure to the fracture, which hinders fracture reduction and internal fixation [10]. This article retrospectively analyzes the clinical data of 15 adolescents and explores the effects of open reduction through the anterior approach and Herbert screw fixation for the treatment of capitellar fractures in adolescents.

## Patients and methods

Approval for the retrospective case series was granted by the Institutional Review Board of Children’s Hospital of Chongqing Medical University. Eighteen children and adolescents with capitellar fracture were treated from May 2014 to May 2019 at the Children’s Hospital of Chongqing Medical University. The inclusion criteria were as follows: (1) patients with complete medical record data; (2) patients with coronal fractures of the capitellum confirmed by CT and three-dimensional reconstruction of the affected elbow joint; (3) patients undergoing open reduction and Herbert screw fixation through transverse incision in the cubital crease via the anterior approach; and (4) patients who completed a follow-up of at least 12 months. Two of these 18 patients were diagnosed with nondisplaced fractures and treated nonoperatively with long arm cast immobilization. One patient was lost to follow-up 8 weeks after the operation. These three patients were excluded from this study. The other 15 patients had adequate clinical and radiographic follow-up and were included in the study.

Under successful intravenous and inhalation anesthesia, the patient was placed in the supine position with a pneumatic tourniquet applied to the affected limb. A transverse incision was made at the cubital crease on the anterior aspect of the elbow (the midpoint of the incision was at the lateral 1/3 of the cubital crease) to expose the fracture site via the intermuscular space, with protection of the radial nerves between the brachialis and brachioradialis. Under direct vision, reduction of the capitellum was performed. A Kirschner wire was used to temporarily fix the fractured fragment. A Herbert screw was inserted over the Kirschner wire after C-arm fluoroscopy showed satisfactory reduction. Then, C-arm fluoroscopy was repeated to confirm the effectiveness of the reduction. The incision was closed with absorbable suture, and the affected limb was immobilized with a long arm plaster slab. The incision dressing was changed 2–3 days after surgery. One week after the operation, the cast was removed and replaced with an orthosis. Elbow function training started under the guidance of a physician at the first outpatient review, usually 2 to 3 weeks after surgery.

The follow-up was performed every 2–3 weeks after surgery until the fracture line was blurred or disappeared and the elbow joint was painless during functional activities. Then, the follow-up was performed every 2–3 months within 1 year after surgery and every 6 months afterwards. The degree of pain in the affected limb, daily activities, and elbow function were examined and recorded. Elbow function was assessed according to the Mayo Elbow Performance index (MEPI) [11] and the Broberg-Morrey rating system [12]. A score ≥ 90 on the MEPI was considered excellent; scores from 75 to 89 were considered good; scores from 60 to 74 were considered fair; and scores < 60 were considered poor. A Broberg-Morrey score ≥ 95 was considered excellent; scores from 80 to 94 were considered good; scores from 60 to 79 were considered fair; and scores ≤ 59 were considered poor.

## Results

A total of 15 adolescents were enrolled in this study, including 10 boys and 5 girls with an average age of 13.1 years (range, 11 to 15 years) at the time of surgery. Fractures were in the left elbow in six patients and in the right elbow in nine patients. The mean time to surgical intervention was 3.7 days (range, 1 to 9 days) (as shown in Table 1). All operations were performed by the same senior surgeon. The operative time was approximately 35–60 min. Blood loss was approximately 10–40 ml, and no vascular or nerve damage was noted during the operation. After the operation, the sensation and motion of the patients’ fingers were normal.

**Table 1:** Patient information and functional outcomes

Patient ID	Age	Sex	Injured sites	Causes of injury	Time from injury to surgery	Time for final follow-up (months)	Extension on the affected side	Flexion on the affected side	Pronation on the affected side	Supination on the affected side	BM score	MEPI	Complications
1	13	G	L	Fall	3	12	0	135	90	90	100	100	
2	13	B	R	Fall during running	5	12	0	140	90	80	100	100	
3	15	B	L	Fall during a basketball game	5	12	− 30	120	60	70	86	80	Elbow dysfunction
4	12	B	L	Fall	9	15	− 20	130	70	80	90	85	Elbow dysfunction
5	15	B	R	Fall during running	1	15	0	140	80	80	100	100	
6	11	G	R	Fall	3	15	− 20	100	60	50	75	85	Elbow dysfunction
7	14	G	L	Fall	3	17	0	140	70	90	100	100	
8	13	B	R	Fall	3	18	0	140	90	90	100	100	
9	13	B	L	Fall	3	18	0	135	90	80	100	100	
10	13	B	R	Fall during running	4	19	− 5	135	80	90	99	100	
11	12	B	R	Fall	2	23	0	135	90	90	100	100	
12	13	B	R	Fall	3	24	0	130	90	90	99	100	Incision scar
13	14	B	L	Fall during running	4	26	− 5	140	90	90	100	100	
14	14	G	R	Fall from a bicycle	4	27	0	140	90	90	100	100	
15	12	G	R	Fall	4	36	0	135	80	80	100	100	

The patients were followed up for an average of 19.2 months (12–36 months). No signs of infection, such as redness, swelling, or exudation were observed at the incision after surgery. The incision had healed by the outpatient follow-up 2–3 weeks after the operation. One patient had an incision scar. The fracture healing time was 4–7 weeks (6 weeks on average). No delayed union or nonunion of the fracture was noted.

**Fig. 1:**
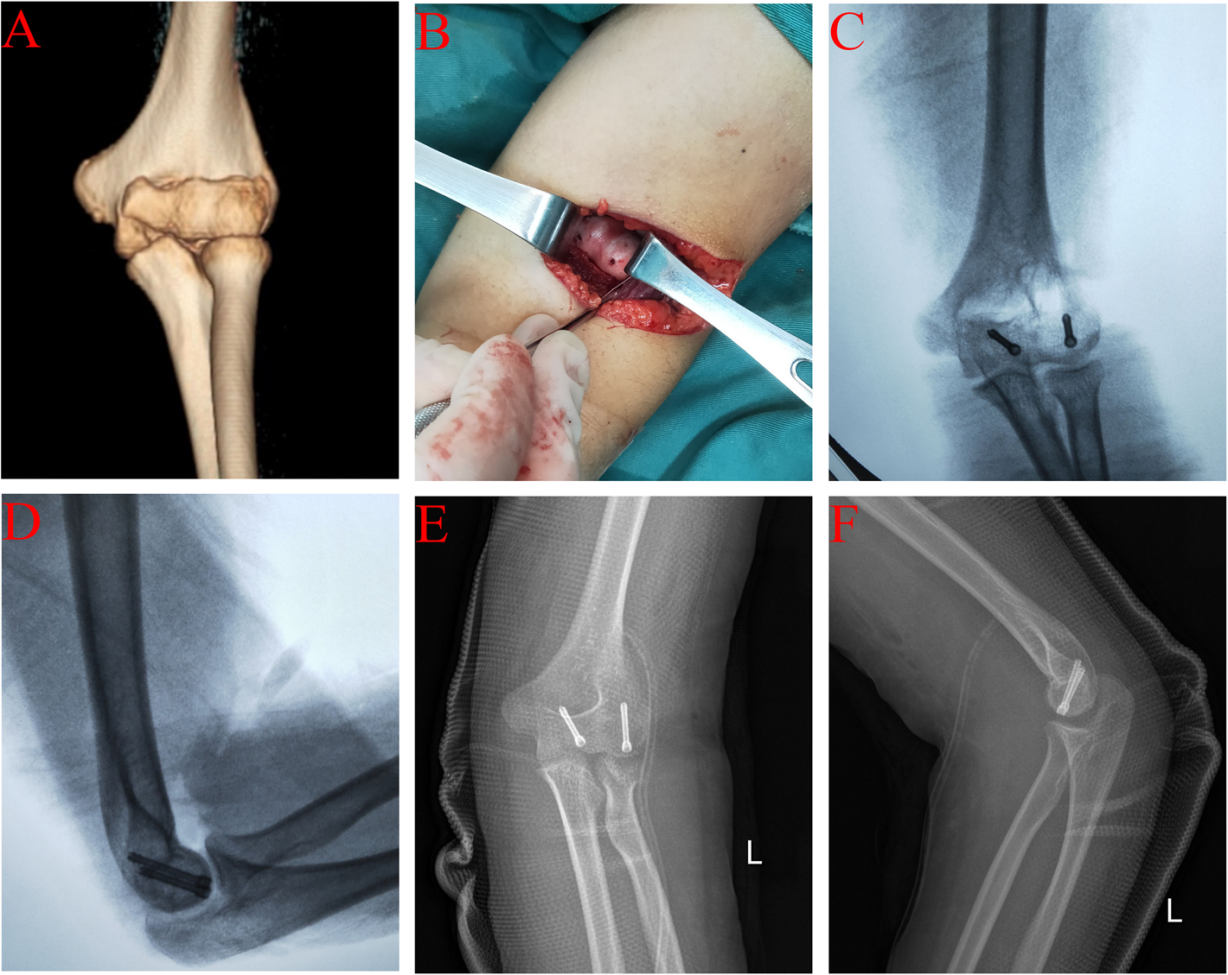
A 13-year-old girl presented with swelling, pain, and dysfunction of the left upper limb due to a fall. CT of the elbow joint showed a capitellar fracture (**A**). She underwent reduction of the capitellar fracture via the anterior approach under direct vision. Herbert screws were used to fixate the fracture fragments (**B**). C-arm fluoroscopy (**C** anteroposterior view of the elbow joint; **D** lateral view of the elbow joint) confirmed satisfactory reduction and fixation of the fractures. Follow-up radiography was performed on the first day after surgery. The anteroposterior view (**E**) and lateral view (**F**) showed that the reduction and fixation were good

At the last follow-up, the internal fixation was stable, and no breakage or loosening of screws, infection, posttraumatic arthritis, or other complications were reported. The average ranges of extension, flexion, pronation, and supination of the affected elbow joint were − 5.3° (range, − 30–0°), 133° (range, 100–140°), 81.3° (range, 60–90°), and 82.7° (range, 50–90°), respectively (as shown in Table 1). The mean MEPI score was 96.7 points (range 80–100 points). The MEPI scores indicated excellent efficacy in 12 patients and good efficacy in three patients. The mean Broberg-Morrey score was 96.6 points (range 75–100 points). By this score, the efficacy was excellent in 12 patients, good in two patients, and fair in one patient. A typical case is shown in Fig. 1.

A total of three patients developed elbow dysfunction after surgery. Patient 3 was a 15-year-old boy who was followed up by another hospital after the operation. The affected elbow was immobilized with a cast, and he did not perform functional exercise. At the 4th week after the operation, he was evaluated in the outpatient clinic of our hospital for the first time and started functional training. The patient started elbow functional exercises (physical therapy) in the department of rehabilitation of our hospital after the flexion contracture of his elbow joint was approximately 40° 4 months after the operation. The range of flexion and extension of his elbow joint reached 30–120° at the last follow-up 15 months after surgery. Patient 4 had missed diagnosis by another hospital. The patient was diagnosed with capitellar fracture on CT in our hospital 1 week after the injury and received surgery on the 9th day after the injury. After the cast was removed on the 6th day after the operation, the patient started functional exercise. At the last follow-up 12 months after surgery, the patient had a range of elbow motion of 20–130°. Patient 6 was an 11-year-old girl who did not follow the doctor’s instructions for functional exercise due to the fear of pain. At the last follow-up 15 months after the operation, radiographs indicated that the fracture had healed well, but the range of elbow motion was limited, with ranges of flexion and extension of 20–100°.

## Discussion

Capitellar fractures in children are more common among adolescents than other age groups and are related to the development of the anatomical structure of the elbow joint [8, 13]. In young children, anatomical and mechanical weak areas exist at the junction of the humeral condyle and the humeral shaft [14]. Trauma often leads to humeral supracondylar fractures. In addition, nonossified secondary ossification centers in children’s elbow joints, which starts to fuse around the age of 12 years [15], provide a cushioning effect against external stress. Therefore, capitulum fractures often occur in older children. The ossification center of the capitellum has an anterior inclination of approximately 40°, resulting in an angle of approximately 140° between the capitellum and the humeral shaft [16]. Capitellar fracture is often considered to be the result of the shear force on the capitellum from the direct impact of the radial head on the capitellum [17, 18]. Therefore, coronal fractures of the capitellum are the most common type.

Controversies remain regarding the surgical approach for capitellar fractures. Many researchers have utilized the lateral or posterior approach in their studies [1, 5, 8]. Ravishankar et al. [19] suggested that the lateral approach may cause laxity of the lateral collateral ligament, and the repair of the ligament during the operation will prolong the operation time. Ballesteros et al. [10] suggested that due to the lack of operating space when using the lateral approach, the screw cannot be implanted perpendicularly to the fracture surface, which affects the fixation effect. The posterior approach may damage the blood supply to the posterior capitellum [20, 21]. The anterior approach can provide sufficient exposure of the fractured fragments and has gradually become the preferred surgical approach for open reduction of supracondylar fractures of the humerus in children [22, 23]. However, some researchers argue that this approach may damage blood vessels and nerves [24]. In this study, capitellar fractures were reduced and fixed via the anterior approach. Novessel or nerve damage occurred during the operation. Since the median nerve, ulnar nerve, and brachial vessels are medial to the elbow joint, damage to the main blood vessels and nerves can be avoided with slight retraction of the superficial elbow vein and cutaneous nerve after incision and protection of the radial nerves between the brachialis and brachioradialis.

Moreover, due to the non-fully developed muscles of the anterior elbow in children, the fracture site can be quickly and clearly exposed by gentle retraction of muscles during the operation, and the fracture can be reduced and fixated under direct vision. In addition, because the incision is made along the cubital crease of the elbow, which is consistent with the skin pattern of the elbow joint, the application of intradermal suture is conducive to incision healing and an aesthetic outcome. Only one patient had an incision scar in this study. The results of this study show that the anterior approach is safe for the surgical treatment of capitellar fractures in children and can sufficiently expose the operative field to allow the surgeon to perform fracture reduction under direct vision.

Choosing an appropriate internal fixator is key to the treatment of capitellar fractures. Kirschner wires are the most commonly used and the best device to stabilize the fracture in younger children, but they have certain limitations for the treatment of capitellar fractures. The Kirschner wire passes through the articular surface but cannot provide stable fixation of the fractured fragments, and long-term cast immobilization after the operation is needed, which precludes early elbow movement and is prone to cause elbow stiffness [25]. Some researchers choose absorbable screws to fix capitellar fractures in children, but their absorbability renders the strength of such screws uncertain. Compared with metal internal fixators, absorbable screws may extend the time needed for external fixation [9]. Capitellar fractures are intra-articular fractures, and long-term external fixation is not conducive to the recovery of elbow function. Moreover, an absorbable material may cause serious complications such as foreign body reactions, and a potential risk for stunted development of the epiphysis exists in children [26].

In this study, Herbert screws were used to fix capitellar fractures. Elkowitz et al. [27] suggested that headless screws provide better stability than cancellous bone screws. For the treatment of intra-articular fractures, anatomical reduction and firm fixation of the fracture are key to fracture healing and functional recovery [28]. Since the Herbert screw is completely driven into the articular cartilage, it will not affect the smoothness or integrity of the articular surface and has almost no effect on joint movement. However, the joint surface was still damaged by the screws, and although no elbow arthritis was observed in this study, a long-term follow-up is still needed. Herbert screws can provide firm fixation of fracture fragments, and patients can start functional exercise early after surgery, which can effectively prevent postoperative complications such as elbow stiffness. However, early functional exercise may be associated with a risk of fracture displacement or internal fixation failure. Therefore, functional exercises must be started gradually under the guidance of a physician.

Some questions remain regarding the Herbert screw. Because the screw is completely buried in the cartilage, precluding surgical removal, it will remain in the bone permanently. A postoperative infection would be catastrophic; however, considering that most of these fractures are closed fractures, the risk of infection is low. In this study, no infections occurred in any patients but vigilance is still needed. If postoperative infection occurs, surgical removal of the internal fixation may be the best strategy. In addition, the management of a possible future fracture of the distal humerus would be more complicated due to the presence of the screw. Since the patients were all adolescents, the risk of refracture was not significant. If surgical treatment is necessary, the internal fixation can be removed at the same time.

Three patients in this study had elbow dysfunction, one of whom had a missed diagnosis in another hospital and was later diagnosed with capitellar fracture by CT examination in our hospital. Due to the long time from injury to surgery, the child still had a certain degree of elbow dysfunction even after functional exercises. The other two adolescents developed postoperative elbow dysfunction due to nonadherence to functional exercises. The function of the elbow joint in the other adolescents had recovered well at the last follow-up. We should be wary of capitellar fractures in adolescents with elbow joint injuries. CT, MRI, or arthrography can be used to confirm the diagnosis. For those with surgical indications, surgery should be performed as soon as possible, and effective functional exercises should be carried out promptly after the operation. These measures can effectively prevent postoperative elbow dysfunction.

The limitation for this retrospective study is the lack of a randomized controlled design. As capitellar fractures are rare, the sample was small, and long-term follow-up was not performed. Future research should include a multicentre, randomized, controlled study to further investigate the effectiveness (and its correlates) of open reduction through the anterior approach and the use of Herbert screws in the treatment of capitellar fractures in adolescents.

## Conclusion

In this study, open reduction through the anterior approach and Herbert screw fixation were used to treat coronal fractures of the capitellum in adolescents. The results of the study show that the anterior approach is safe for the surgical repair of capitellar fractures in adolescents and can sufficiently expose the operative field for the surgeon to perform fracture reduction under direct vision. Herbert screw fixation can provide reliable fixation without damaging the flatness of the articular surface. Open reduction via the anterior approach with Herbert screw fixation is a feasible surgical method for the treatment of coronal fractures of the capitellum in adolescents.

## Data Availability

The datasets used and analyzed during the current study are available from the corresponding author on reasonable request.

## Abbreviations

MEPI: Mayo Elbow Performance index
CT: Computed tomography
MRI: Magnetic resonance imaging
